# Leaching mechanism and behavior of rare earth elements from roasted coal gangue using citric acid

**DOI:** 10.1038/s41598-026-61534-2

**Published:** 2026-07-31

**Authors:** Lei Zhang, Jinhe Pan, Fan Yang, Changchun Zhou, Shulan Shi, Xin He, Bin Dong, Jinpeng Feng

**Affiliations:** 1 Key Laboratory of Coal Processing & Efficient Utilization, Ministry of Education, School of Chemical Engineering and Technology, China University of Mining & Technology, Xuzhou, 221116 China; 2 Inspur Computer Technology Co., Ltd., Ji’nan, 250200 China; 3 School of Resources, Environment and Materials, State Key Laboratory of Featured Metal Materials and Life-Cycle Safety for Composite Structures, Guangxi University, Nanning, 530004 China

**Keywords:** Coal gangue, Rare earth elements, Citric acid, Hydrochloric acid, Leaching, Chemistry, Energy science and technology, Environmental sciences

## Abstract

The recovery of rare earth elements (lanthanides and yttrium, REY) from coal gangue offers a potential approach for the resource utilization of this industrial waste. In this work, the effect of citric acid on the leaching of REY from roasted coal gangue was systematically investigated. The leaching mechanism of REY was elucidated by comparing the leaching behavior of REY in different acid systems, combined with zeta potential analysis and solution chemical equilibrium calculations. The maximum leaching efficiencies of REY, light REY (LREY), and heavy REY (HREY) were 48.6%, 59.1%, and 17.2%, respectively, achieved at 0.8 mol/L citric acid, a liquid-to-solid ratio of 25 mL/g, 150 min, and 90 °C. Acid leaching experiments at a fixed concentration of 0.8 mol/L showed that hydrochloric acid leached REY more rapidly than citric acid. At the same initial pH (approximately 1.5), citric acid achieved a higher REY leaching efficiency, and the pH change of the solution during leaching was the smallest, at 0.43. The citric acid leaching reaction mainly occurs in the pH range of 1.5–2.0. Meanwhile, the Al leaching efficiency was 51.0% under the condition of 0.8 mol/L citric acid. SEM–EDS analysis reveals that the Si/Al atomic ratio on the solid surface increased from an initial value of 1.0 to 2.6 and 3.0 after leaching with 0.8 mol/L citric acid and hydrochloric acid, respectively. Zeta potential analysis and solution chemical equilibrium calculations indicate that the complexation between citrate ions and rare earth ions is weak during the leaching stage. Therefore, the leaching of REY from roasted coal gangue by citric acid is mainly due to the effect of H^+^. These findings can provide support for improving sustainable recovery strategies of REY from coal gangue.

## Introduction

Rare earth elements (lanthanides and yttrium, REY) play important roles in many core fields and have consequently been included in the critical metals list of major countries or organizations such as the United States, China, and the European Union^1–3^. The widening gap between REY demand and supply urgently requires stable and abundant alternative sources^3,4^. Coal has been regarded as one of the most promising REY sources following the discovery of REY-rich coals in the United States^5,6^, China^7–10^, and Russia^11,12^. Among previous studies^13–16^, a consensus has been established that REY in coal is primarily hosted within inorganic minerals rather than organic matter. In other words, REY tends to be preferentially enriched in coal gangue during coal mining and washing. Consequently, the recovery of REY from coal gangue has attracted the attention of researchers^17–19^.

Coal is one of the main energy sources in many countries, with a global consumption of 9241.5 million tons in 2024, accounting for 27.9% of the world’s total energy supply^20^. Coal gangue is a solid waste generated from coal mining and preparation processes, the output of which is approximately 15–20% of the total coal production^21^. The cumulative amount of coal gangue has increased significantly with coal mining, making it one of the largest industrial wastes^22,23^. Coal gangue not only occupies large amounts of land, but also causes widespread environmental concerns from the release of toxic heavy metals^24^. During the accumulation of coal gangue, spontaneous combustion induced by oxidation can readily occur, resulting in the emission of hazardous gases such as SO_2_, NO_x_, and CO_x_^25^. Therefore, the proper disposal and even comprehensive utilization of coal gangue is a pressing task that requires immediate attention. Recovering REY from coal gangue not only holds promise for alleviating supply pressure on conventional rare earth resources but also promotes the high-value utilization of coal gangue.

In view of the modes of occurrence of REY and characteristics of coal gangue, acid leaching is the necessary process for REY recovery from coal gangue^14,26–28^. Sequential chemical extraction reveals that REY in coal gangue primarily occurs in stable aluminosilicate forms^28–30^. Through microscopic observation using a scanning electron microscope coupled with an energy dispersive spectrometer (SEM–EDS), Chen et al.^17^ identified micro-particles of bastnaesite and monazite in coal gangue from the Haerwusu open-pit mine in Jungar coalfield, which were embedded within the kaolinite matrix. Similarly, Wang et al.^27^ also confirmed via SEM–EDS analysis that REY in coal gangue from the Jungar coalfield was encapsulated within silicate minerals. Before acid leaching, roasting is a commonly used activation pretreatment method that effectively disrupts the crystal structure of minerals in coal gangue, thereby improving the leaching recovery of REY^17,30,31^. Among all leaching parameters, the type of acid is one of the most important factors affecting the efficiency and cost of the process. Inorganic acids are considered the most effective leaching agents due to their strong acidity. The large amount of hydrogen ions (H^+^) provided by inorganic acids during the leaching process can effectively disrupt the structure of roasted coal gangue and release the REY contained within it^17,29^. However, inorganic acids are strongly corrosive to equipment, and the acid in the waste solution is difficult to recover, which makes them more likely to cause environmental problems.

In recent years, numerous studies have employed organic acids to extract REY from coal-based solid waste^32–35^, owing to their environmental friendliness. Banerjee et al.^34^ used organic carboxylic acids to leach REY from coal ash. The leaching efficiency of REY varied significantly among the different organic acids, with tartaric acid showing the highest efficiency, followed by lactic acid, citric acid, malonic acid, and succinic acid. For the roasted product of coal coarse refuse, citric acid and DL‑malic acid exhibited the best leaching performance for REY^32^. Zeta potential analysis and antisolvent precipitation experiments indicate that the complexation of REY by citric acid can improve their leaching recovery from coal fly ash^33^. Gao et al.^36^ used citric acid instead of hydrochloric acid to extract REY from coal fly ash activated by sodium carbonate roasting, achieving a REY leaching efficiency of 71.5% at a citric acid concentration of 0.5 mol/L. Currently, citric acid has shown unique advantages in the green leaching of REY from coal fly ash. However, studies on its application for REY recovery from coal gangue remain limited. The occurrence mode of REY in coal gangue differs fundamentally from that in coal fly ash, which means the leaching behavior of REY from coal gangue cannot simply be understood through existing knowledge of coal fly ash.

Therefore, this paper systematically investigated the effect of citric acid on the leaching of REY from coal gangue. Before leaching, the coal gangue was subjected to roasting pretreatment to disrupt the stable aluminosilicate mineral structure and enhance the leachability of REY^17^. To elucidate the leaching behavior of REY, hydrochloric acid leaching experiments were conducted for comparison. The mechanism of REY leaching from roasted coal gangue by citric acid was investigated through morphological characterization of leaching residue, electro-kinetic tests, and solution chemical equilibrium calculations. This research can provide valuable insights for the design of green and sustainable processes for extracting REY from roasted products of coal gangue.

## Materials and methods

### Materials

The sample in this study was coal gangue collected from Haerwusu mine in the Jungar coalfield, Inner Mongolia, China, where coal is rich in REY^37,38^. The collected sample was divided into two portions. One portion was stored as a reserve, while the other portion was pulverized and completely passed through a standard sieve with a mesh size of 0.075 mm. The powder sample was dried to constant weight in a forced-air drying oven at 105 °C and subsequently used for the roasting-leaching experiments. Analytical-grade citric acid monohydrate and hydrochloric acid, both purchased from Sinopharm Chemical Reagent Co., Ltd., were used for the leaching experiments. Ultrapure water (resistivity 18.2 MΩ·cm) was used throughout the experiments to prevent the introduction of impurities.

**Fig. 1:**
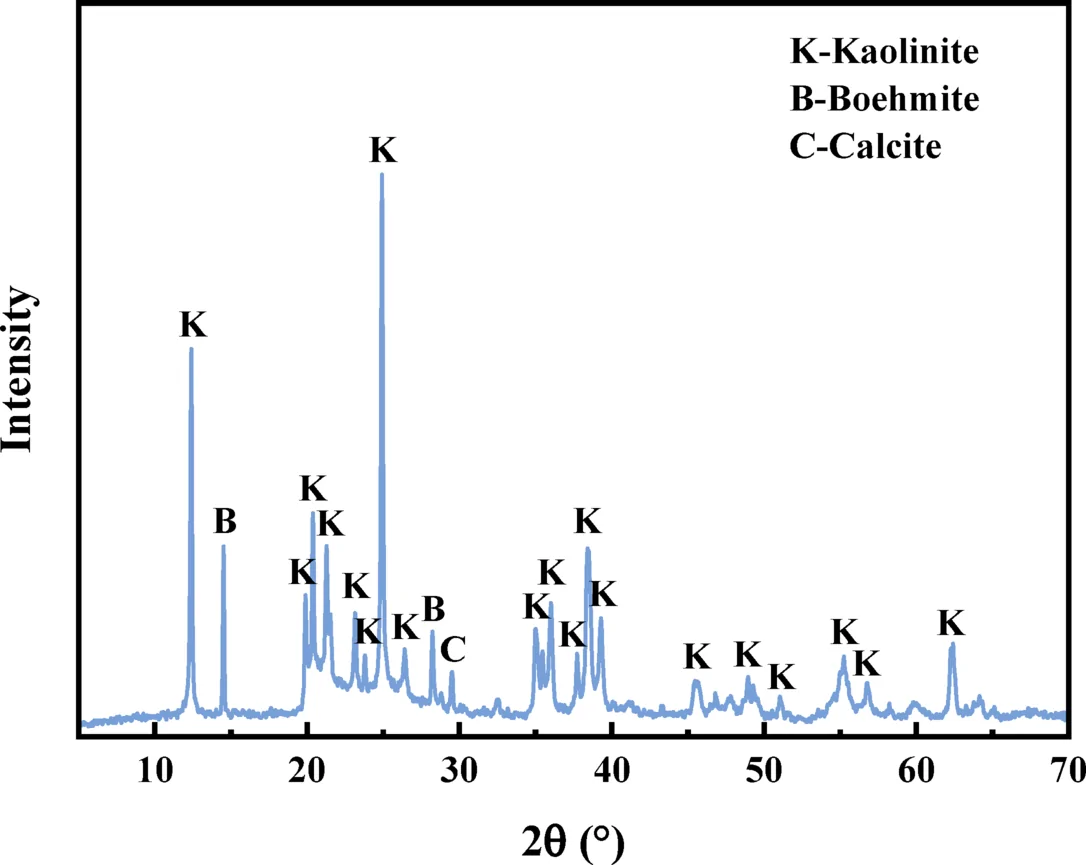
XRD patterns of raw coal gangue.

The main components of this coal gangue sample were Si and Al, with SiO_2_ and Al_2_O_3_ contents of 34.65% and 36.63% (Table 1), respectively. The loss on ignition (LOI) was 26.05%, which indicates that this coal gangue contains a certain amount of organic matter. X-ray diffraction (XRD) analysis indicates that this coal gangue was primarily composed of kaolinite (Fig. 1), which is consistent with its main chemical composition. Additionally, coal gangue also contained small amounts of boehmite and calcite. The REY content in this coal gangue was 175.92 mg/kg, which exceeds the average level of REY in Chinese coals^39^. Among the REY, Ce had the highest content at 57.31 mg/kg, accounting for 32.58% of the total. In addition, REY are usually classified by their unique physicochemical properties into light REY (LREY, including La, Ce, Pr, Nd, Sm, and Eu) and heavy REY (HREY, including Gd, Tb, Dy, Ho, Er, Tm, Yb, Lu, and Y). The LREY and HREY contents in this coal gangue were 129.37 mg/kg and 46.55 mg/kg, respectively. In our previous work, microscopic observation by SEM–EDS revealed that monazite and bastnaesite are the main carrier minerals of REY in this coal gangue, and they are embedded within kaolinite^17,40^. Roasting treatment can expose the REY‑bearing minerals encapsulated in kaolinite. Meanwhile, bastnaesite decomposes at high temperatures into rare earth oxides and rare earth fluorides. These changes are beneficial for the subsequent leaching of REY.

**Table 1:** Chemical compositions and rare earth elements contents of coal gangue.

Compositions	SiO_2_	Al_2_O_3_	Fe_2_O_3_	CaO	TiO_2_	K_2_O	S	P	LOI
Content (%)	34.65	36.63	0.28	1.25	0.74	0.16	0.15	0.09	26.05
Elements	La	Ce	Pr	Nd	Sm	Eu	Gd	Tb	Dy
Content (mg/kg)	34.56	57.31	7.86	25.60	3.22	0.82	4.48	0.85	5.46
Distribution (%)	19.65	32.58	4.47	14.55	1.83	0.47	2.55	0.48	3.10
Elements	Ho	Er	Tm	Yb	Lu	Y	REY	LREY	HREY
Content (mg/kg)	1.07	3.23	0.46	2.92	0.42	27.66	175.92	129.37	46.55
Distribution (%)	0.61	1.84	0.26	1.66	0.24	15.72	100.00	73.54	26.46

### Experimental procedures

The coal gangue was roasted at 600 °C in a muffle furnace for 2 h to remove organic matter and activate inorganic minerals for high REY recovery, as reported in previous research^29^. To evaluate the effect of citric acid on REY leaching recovery from roasted coal gangue, 2.5 g of the sample was placed in a beaker, and then citric acid solution was added at a specified liquid-to-solid ratio (mL/g). The mixture was magnetically stirred at 220 r/min for a predetermined time at a given temperature. The experimental parameters evaluated in this study included citric acid concentration (0.2, 0.4, 0.6, 0.8, 1, 2, and 3 mol/L), liquid-to-solid ratio (5, 10, 15, 25, 50, 75, and 100 mL/g), leaching time (30, 60, 90, 120, 150, 180, and 210 min), and leaching temperature (25, 45, 60, 75, and 90 °C). After leaching, the slurry was filtered using a 0.45 μm hydrophilic polyvinylidene fluoride membrane with the assistance of a pump. The solid residue was dried in a vacuum oven at 60 °C for 24 h for subsequent characterization. The concentrations of REY and Al in the filtrate were determined using inductively coupled plasma-mass spectrometry (ICP-MS, iCAP RQ, Thermo Fisher Scientific) and inductively coupled plasma-optical emission spectrometry (ICP-OES, Optima 8300, PerkinElmer), respectively.

Hydrochloric acid was used as the leaching agent for the inorganic acid leaching of roasted coal gangue. The leaching experiments were carried out at a fixed liquid-to-solid ratio of 25, a temperature of 90 °C, and a stirring rate of 220 r/min. The concentrations of hydrochloric acid were 0.8 mol/L and 0.032 mol/L, respectively, with leaching times ranging from 10 to 210 min. The leaching efficiencies of REY and Al from roasted coal gangue were calculated using Eq. (1).

1 $$\eta = \frac{{C_{L} \times V}}{{C_{S} \times m}} \times 100\%$$

where η is the leaching efficiency of the target element (%), *C*_*L*_​ is the concentration of the element in the final leachate (mg/L), *V* is the total volume of the leachate (L), *C*_*S*_ is the concentration of the element in the roasted coal gangue (mg/kg), and *m* is the mass of the roasted coal gangue used in the leaching experiment (g).

### Characterization and analysis methods

The phase composition of the raw coal gangue was analyzed by XRD using a Bruker D8 Advance diffractometer. XRD analysis was performed with Cu Kα radiation at a voltage of 40 kV and a current of 30 mA. The 2θ scanning range was set to 5°–70°, with a step size of 0.02° and a scanning speed of 5°/min. The contents of major elements in the raw coal gangue were determined by X-ray fluorescence spectrometry (XRF, S8 TIGER, Bruker AXS). The coal gangue was dried at 105 °C to constant weight and then weighed. The dried sample was subsequently roasted at 800 °C until a constant weight was reached. LOI was calculated by the mass difference. After microwave digestion of raw coal gangue, the REY concentration was determined using ICP-MS^29^.

The micromorphology and surface elemental composition of the roasted coal gangue and leaching residues were characterized by field-emission scanning electron microscopy (SEM, MAIA3, TESCAN) coupled with energy-dispersive X-ray spectrometer (EDS, Oxford AZtecLive Ultim Max∞170, Oxford Instruments). Prior to testing, the sample particles were uniformly dispersed onto conductive tape and then sputter-coated with gold to improve their conductivity. The zeta potential analysis was conducted using a Zetasizer Nano ZS90 instrument (Malvern Panalytical) on 50 mg samples suspended in ultrapure water or citric acid solutions. The pH of the suspension was adjusted to 1, 2, 3, 4, and 5 by adding NaOH or HCl solutions. The dissociation equilibrium of citric acid and the complexation equilibrium between citrate ions and rare earth ions in the aqueous solution were analyzed using Visual MINTEQ 3.1 software (https://vminteq.com).

## Results and discussion

### Citric acid leaching process of REY from roasted coal gangue

#### Effect of citric acid concentration on REY leaching

**Fig. 2:**
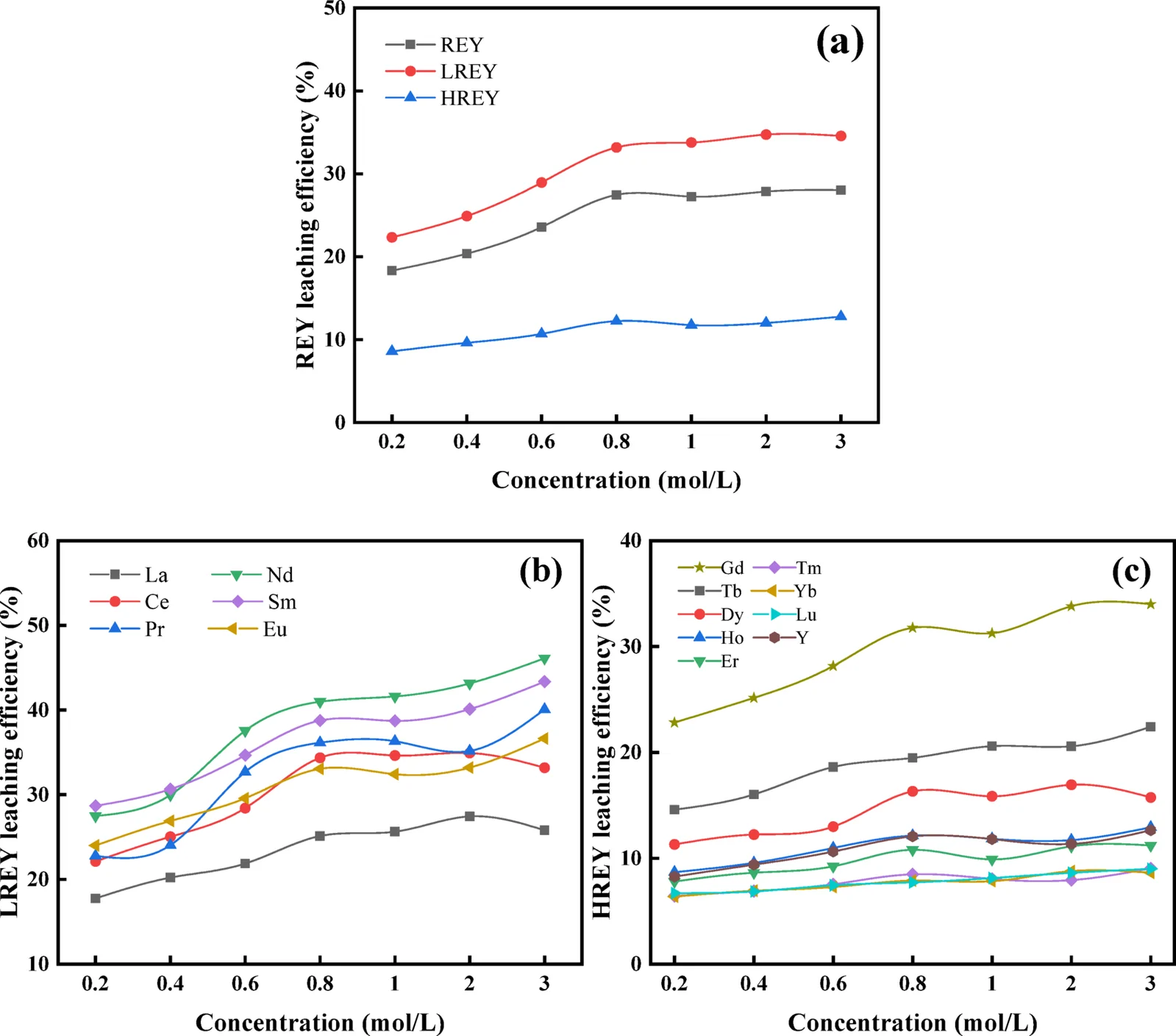
Influence of citric acid concentration on leaching efficiency of rare earth elements from roasted coal gangue.

Citric acid concentration is the primary parameter governing REY leaching performance. Leaching experiments were conducted for 180 min at a liquid-to-solid ratio of 10 and a temperature of 25 °C. The effect of different citric acid concentrations (0.2–3 mol/L) on the leaching efficiency of REY was examined. The results of the leaching experiment are presented in Fig. 2. As the citric acid concentration increased from 0.2 to 0.8 mol/L, the leaching efficiency of REY rose from 18.3% to 27.5%. This is because increasing the citric acid concentration enhances the disruptive effect of H^+^ on the mineral structure, thereby improving the leaching efficiency of REY from roasted coal gangue^32^. When the citric acid concentration exceeded 0.8 mol/L, the leaching reaction of REY from the roasted coal gangue approached equilibrium. Among all REY, the leaching efficiency of Ce was the highest. Overall, the leaching efficiency of LREY was consistently higher than that of HREY, and the improvement in LREY leaching efficiency was also more pronounced. This phenomenon is consistent with the leaching behavior of REY from roasted coal gangue using hydrochloric acid, as reported by Chen et al.^17^.

#### Effect of liquid-to-solid ratio on REY leaching

**Fig. 3:**
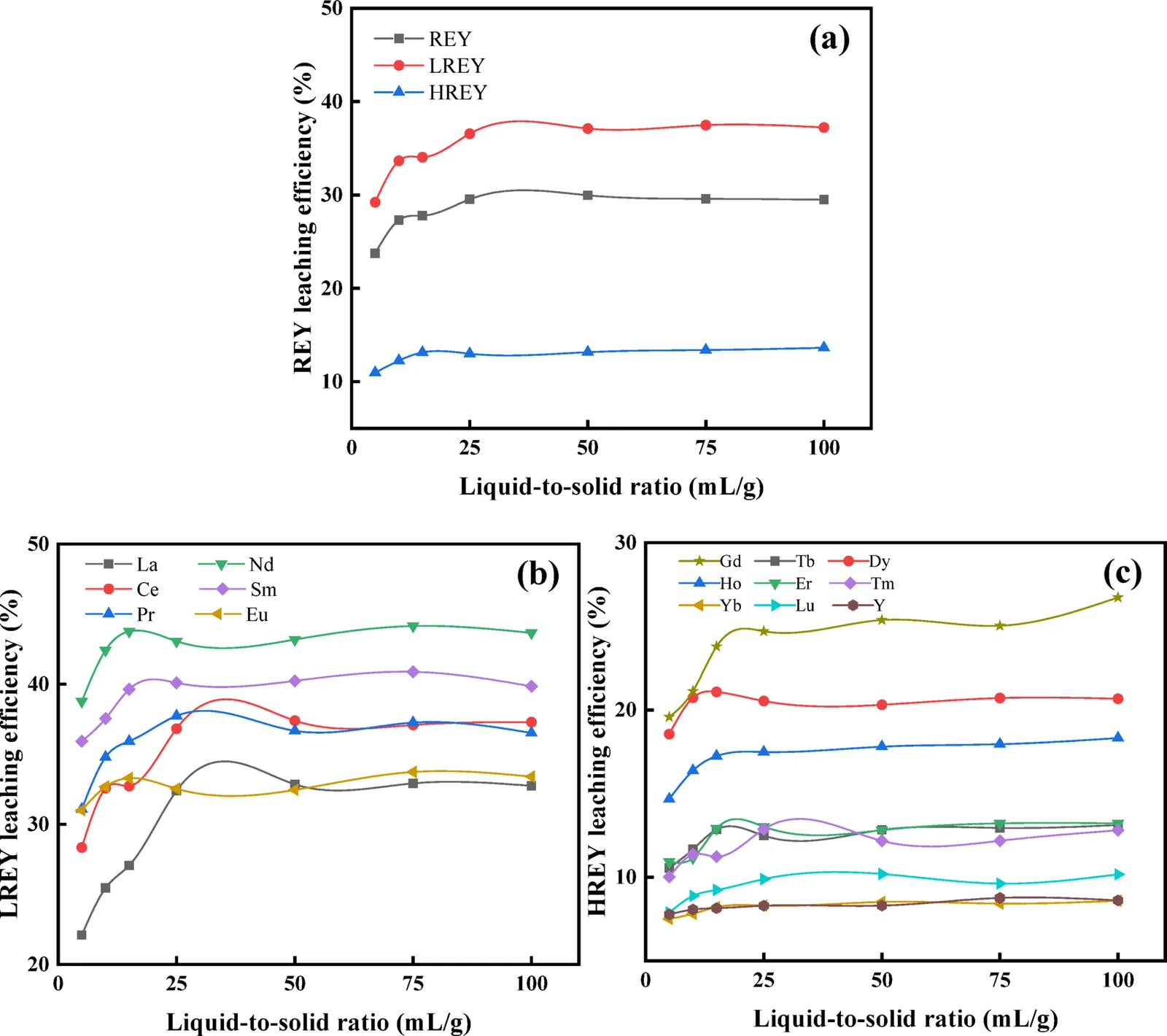
Influence of liquid-to-solid ratio on the leaching efficiency of rare earth elements from roasted coal gangue.

The liquid-to-solid ratio is an important parameter affecting the leaching efficiency, determining the leaching limit of REY and the amount of citric acid consumed. With a fixed citric acid concentration of 0.8 mol/L, leaching time of 180 min, and temperature of 25 °C, the effect of the liquid-to-solid ratio in the range of 5–100 on the leaching efficiency of REY from roasted coal gangue was investigated. As shown in Fig. 3, the REY leaching efficiency increased with increasing liquid-to-solid ratio and leveled off when the ratio reached 25. As the liquid-to-solid ratio increased from 5 to 25, the leaching efficiencies of REY, LREY, and HREY increased from 23.8%, 29.2%, and 11.0% to 29.5%, 36.6%, and 13.0%, respectively. The citric acid leaching process of roasted coal gangue is a solid–liquid reaction, controlled by both surface chemical reaction and ion diffusion^17,41^. An appropriate increase in the liquid-to-solid ratio effectively enlarges the contact area and promotes mass transfer, thereby improving the leaching efficiency of REY. Meanwhile, the lower REY concentration in the leachate facilitates the diffusion of rare earth ions into the solution^33^.

#### Effect of leaching time on REY leaching

**Fig. 4:**
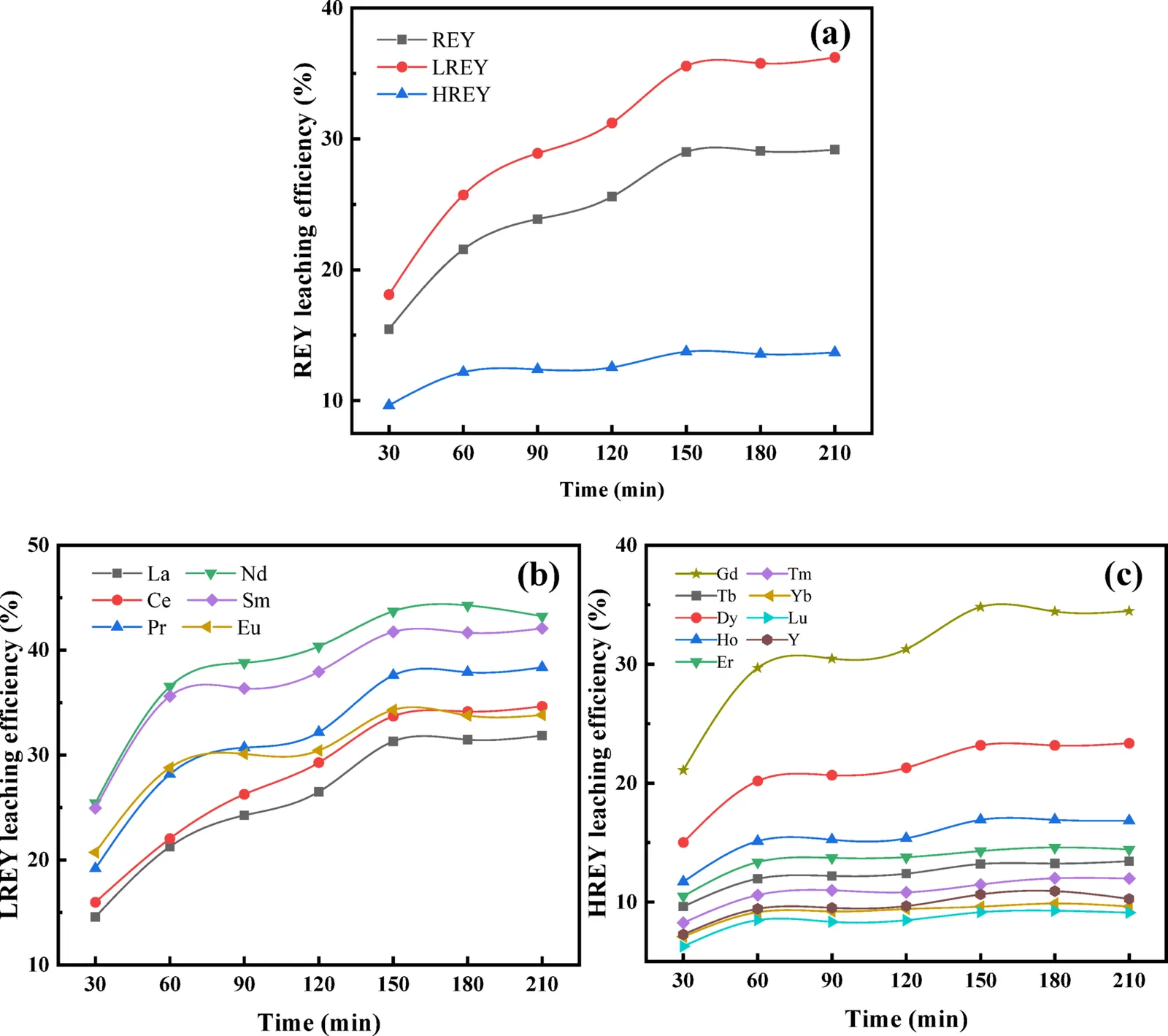
Influence of leaching time on leaching efficiency of rare earth elements from roasted coal gangue.

To investigate the effect of leaching time on the REY leaching efficiency, the experiments were conducted at a leaching temperature of 25 °C, with a citric acid concentration of 0.8 mol/L and a liquid-to-solid ratio of 25. The results of the REY leaching efficiency at different leaching times (30–210 min) are shown in Fig. 4. The overall trend was that the leaching efficiency of REY initially increased and then remained constant as leaching time increased. The leaching process of REY from roasted coal gangue can be divided into three stages. Within the first 60 min of leaching, the leaching efficiencies of all REY increased rapidly. This is mainly because the REY exposed on the surface of the roasted coal gangue are readily leached^27,30^. Specifically, the leaching efficiencies of REY, LREY, and HREY increased to 21.6%, 25.7%, and 12.2%, respectively. From 60 to 150 min, the leaching process may be controlled by internal diffusion through the porous residue layer. Consequently, the magnitude of increase in REY leaching efficiency slowed down relatively. The leaching efficiencies of REY, LREY, and HREY reached 29.0%, 35.6%, and 13.7%, respectively. When the leaching time exceeded 150 min, the leaching reaction of REY nearly ceased, indicating that this process had approached equilibrium.

#### Effect of leaching temperature on REY leaching

**Fig. 5:**
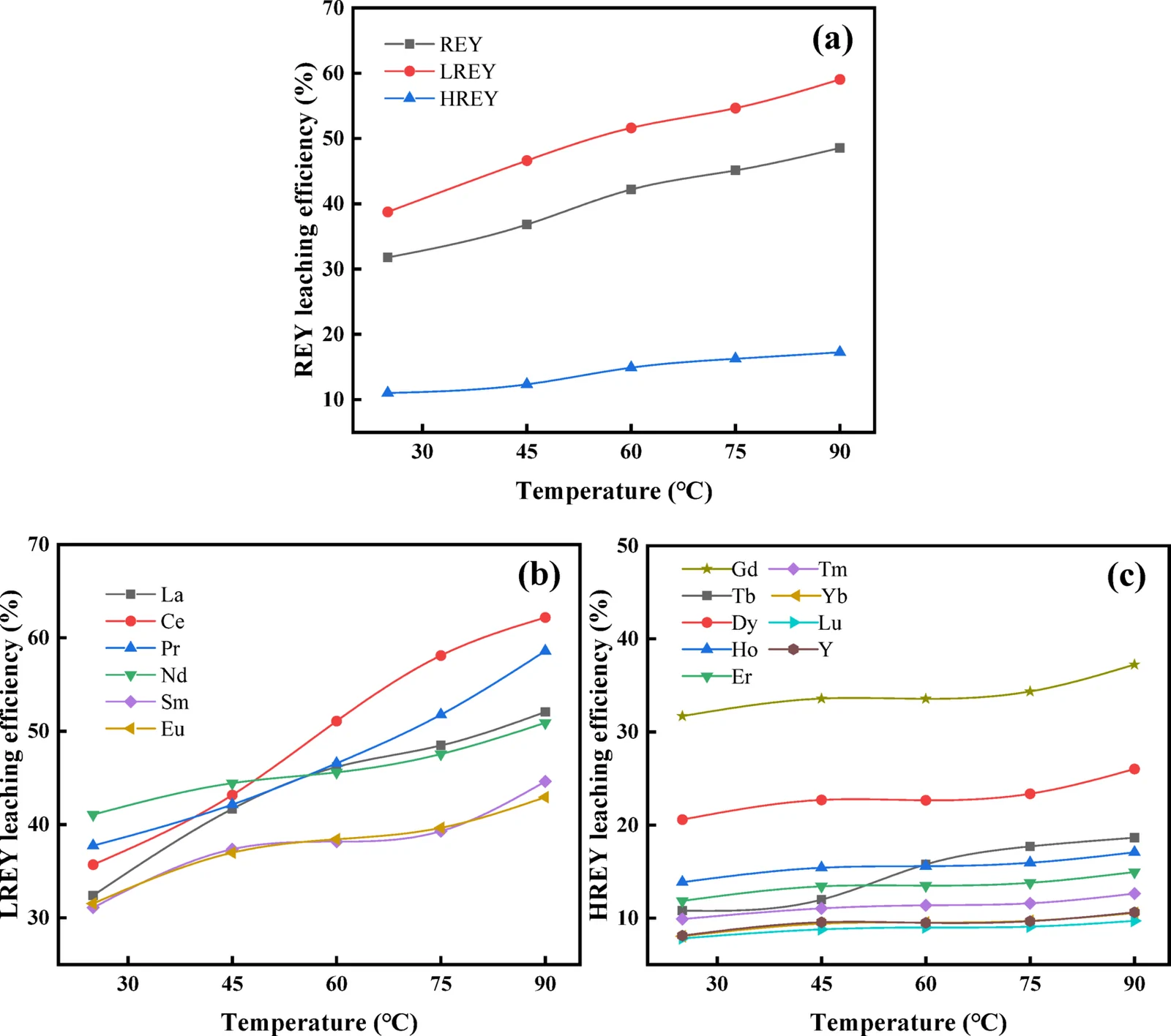
Influence of leaching temperature on the leaching efficiency of rare earth elements from roasted coal gangue.

Among the various parameters of organic acid leaching, temperature is a crucial factor influencing the leaching process. Under fixed conditions of a citric acid concentration of 0.8 mol/L, a liquid-to-solid ratio of 25, and a leaching time of 150 min, the effect of leaching temperatures ranging from 25 to 90 °C on the REY leaching efficiency was investigated. As shown in Fig. 5, the higher the leaching temperature, the higher the REY leaching efficiency. This trend is consistent with that observed when using organic carboxylic acids to leach REY from coal ash^34^. An increase in leaching temperature promotes the ionization of citric acid, thereby increasing the H^+^ concentration in the solution, which facilitates the leaching of REY from roasted coal gangue. When the leaching temperature was increased from 25 to 90 °C, the leaching efficiencies for REY, LREY, and HREY increased from 31.8%, 38.7%, and 11.0% to 48.6%, 59.1%, and 17.2%, respectively. Since further increases in leaching temperature would cause the solution to boil violently, rendering the conditions difficult to control, higher temperatures were not examined in this study. Compared with HREY, the leaching efficiency of LREY increased more significantly. This phenomenon may be related to the different modes of occurrence of HREY and LREY in the coal gangue^17^. As the leaching temperature rose, Ce and Pr exhibited the fastest increase in leaching efficiency, reaching a maximum of 62.2% and 58.6%, respectively.

### Leaching behavior of REY from roasted coal gangue

#### Comparison of citric acid and hydrochloric acid on REY leaching efficiency

**Fig. 6:**
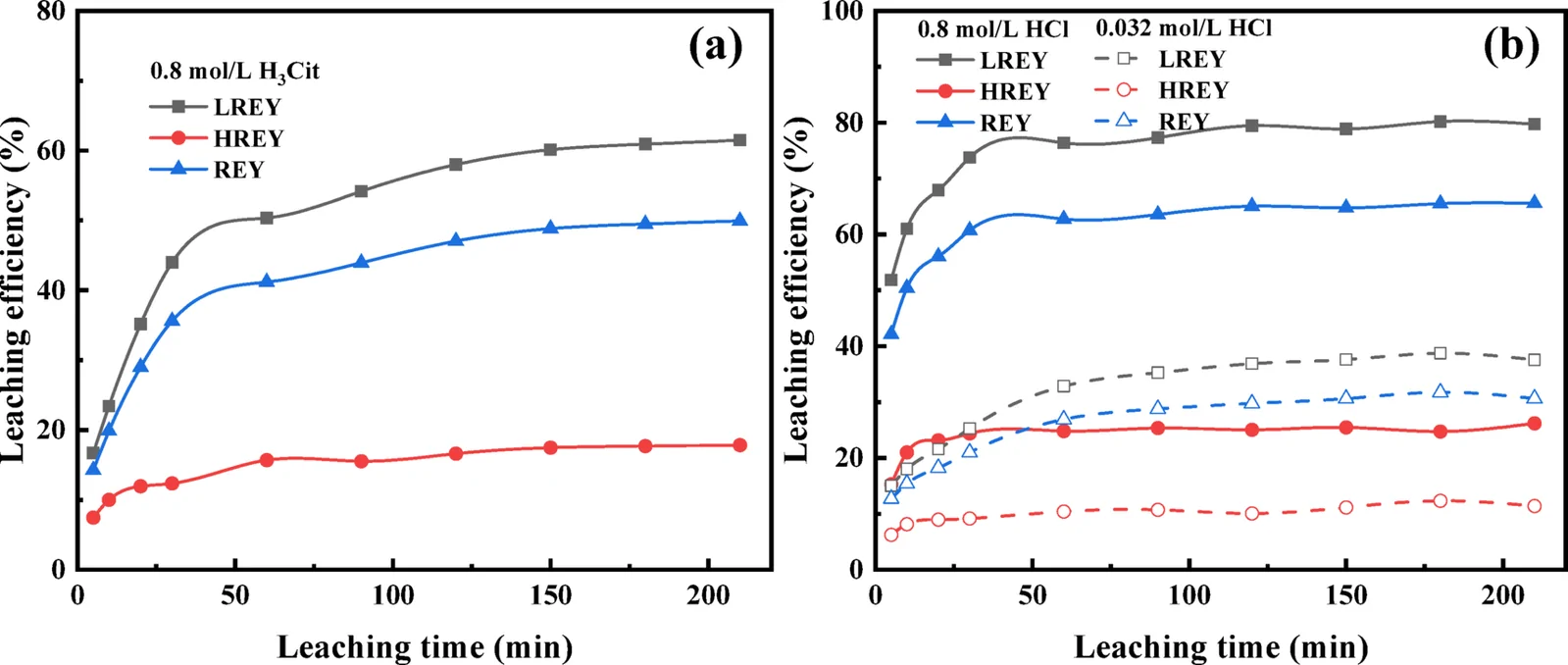
Leaching behavior of rare earth elements as a function of leaching time ((**a**) Citric acid, (**b**) Hydrochloric acid).

The type of acid is considered one of the parameters that most significantly affects the leaching results. In this section, the leaching efficiency of REY from roasted coal gangue using citric acid and hydrochloric acid was compared. All leaching experiments were carried out at a liquid-to-solid ratio of 25 and a leaching temperature of 90 °C, and changes in REY leaching behavior with leaching time are presented in Fig. 6. When a 0.8 mol/L citric acid solution was used as the lixiviant, the REY leaching efficiency increased rapidly within the first 60 min, then increased slowly, and essentially reached a plateau after 150 min. By comparison, when using hydrochloric acid at the same concentration (0.8 mol/L), the REY leaching efficiency rapidly increased in the initial 60 min and then reached equilibrium. The leaching efficiencies of REY, LREY, and HREY reached 62.7%, 76.4%, and 24.8%, respectively, which were significantly higher than the corresponding values in the citric acid system. This may be related to the initial concentration of H^+^ in the solution. Because the initial H^+^ concentration in the hydrochloric acid solution is higher, the REY in the roasted coal gangue is largely leached within a short period of time^17^.

To further distinguish the contribution of the initial H^+^ concentration to the leaching of REY from roasted coal gangue, dilute hydrochloric acid (0.032 mol/L) with an initial pH similar to that of 0.8 mol/L citric acid was selected for comparison. The REY leaching efficiency gradually rose and reached equilibrium at 120 min with 0.032 mol/L hydrochloric acid solution as the leaching agent (Fig. 6b). The final REY leaching efficiency was significantly lower than that of the other two acid systems. This result confirms that low concentrations of H^+^ cannot effectively disrupt the aluminosilicate structure of the roasted coal gangue to release REY. However, citric acid achieved a higher REY leaching efficiency and exhibited different leaching kinetics under the same initial pH conditions. This is mainly attributed to the continuous ionization of citric acid during the leaching process, which provides H^+^ and drives the leaching reaction forward.

**Fig. 7:**
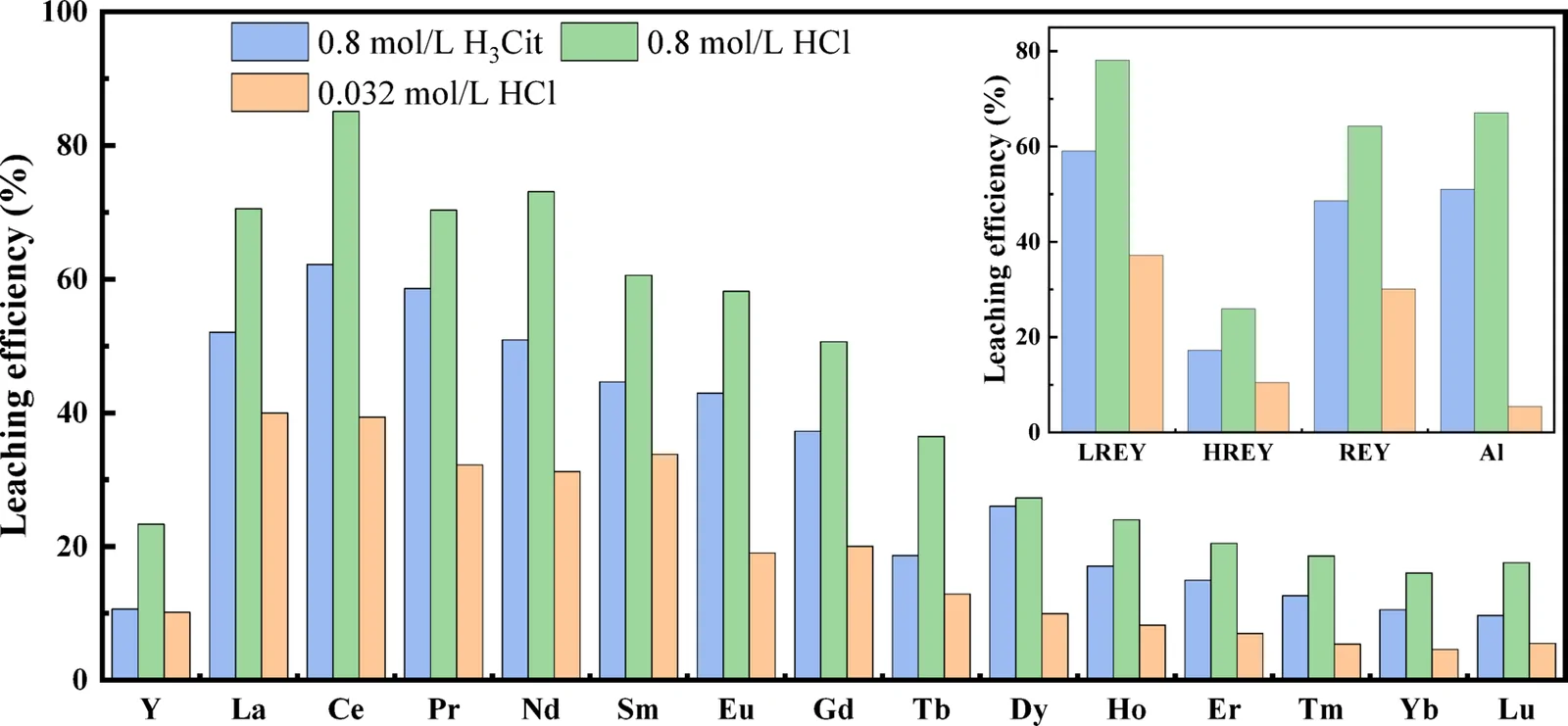
Leaching behavior of rare earth elements and Al from roasted coal gangue under different acid leaching conditions (liquid-to-solid ratio = 25, leaching time = 150 min, leaching temperature = 90 °C).

The leaching efficiencies of individual REY and Al from the roasted coal gangue were compared at a sufficient leaching time of 150 min, as shown in Fig. 7. At a concentration of 0.8 mol/L, hydrochloric acid achieved higher leaching efficiencies for all REY than citric acid, and the improvement was especially significant for LREY. In comparison, the leaching efficiencies of all REY were low using 0.032 mol/L hydrochloric acid (with an initial pH identical to that of 0.8 mol/L citric acid). Meanwhile, the leaching efficiencies of Al at 0.8 mol/L were 51.0% for citric acid and 67.0% for hydrochloric acid, while the minimum Al recovery of 5.4% was observed with 0.032 mol/L hydrochloric acid. This suggests that REY leaching is positively correlated with Al leaching, consistent with the findings of Chen et al.^17^. The pH values of the solutions before and after the leaching of roasted coal gangue, as well as the changes in pH, are shown in Table 2. Among the leaching processes investigated, the pH change was smallest for the 0.8 mol/L citric acid solution, at 0.43. This is attributed to the continuous dissociation of citric acid during leaching, suggesting that the leaching reaction primarily occurs within a pH range of 1.5–2.0. The 0.032 mol/L hydrochloric acid solution showed the largest pH change of 1.95, due to its lowest H^+^ concentration.

**Table 2:** The pH values of the acid solution before and after leaching (25 °C).

Leaching agent	Before leaching	After leaching	Change value
Citric acid solution (0.8 mol/L)	1.58	2.01	0.43
Hydrochloric acid solution (0.8 mol/L)	0.09	0.71	0.62
Hydrochloric acid solution (0.032 mol/L)	1.51	3.46	1.95

#### Surface elemental composition and morphological analysis of leaching residue

**Fig. 8:**
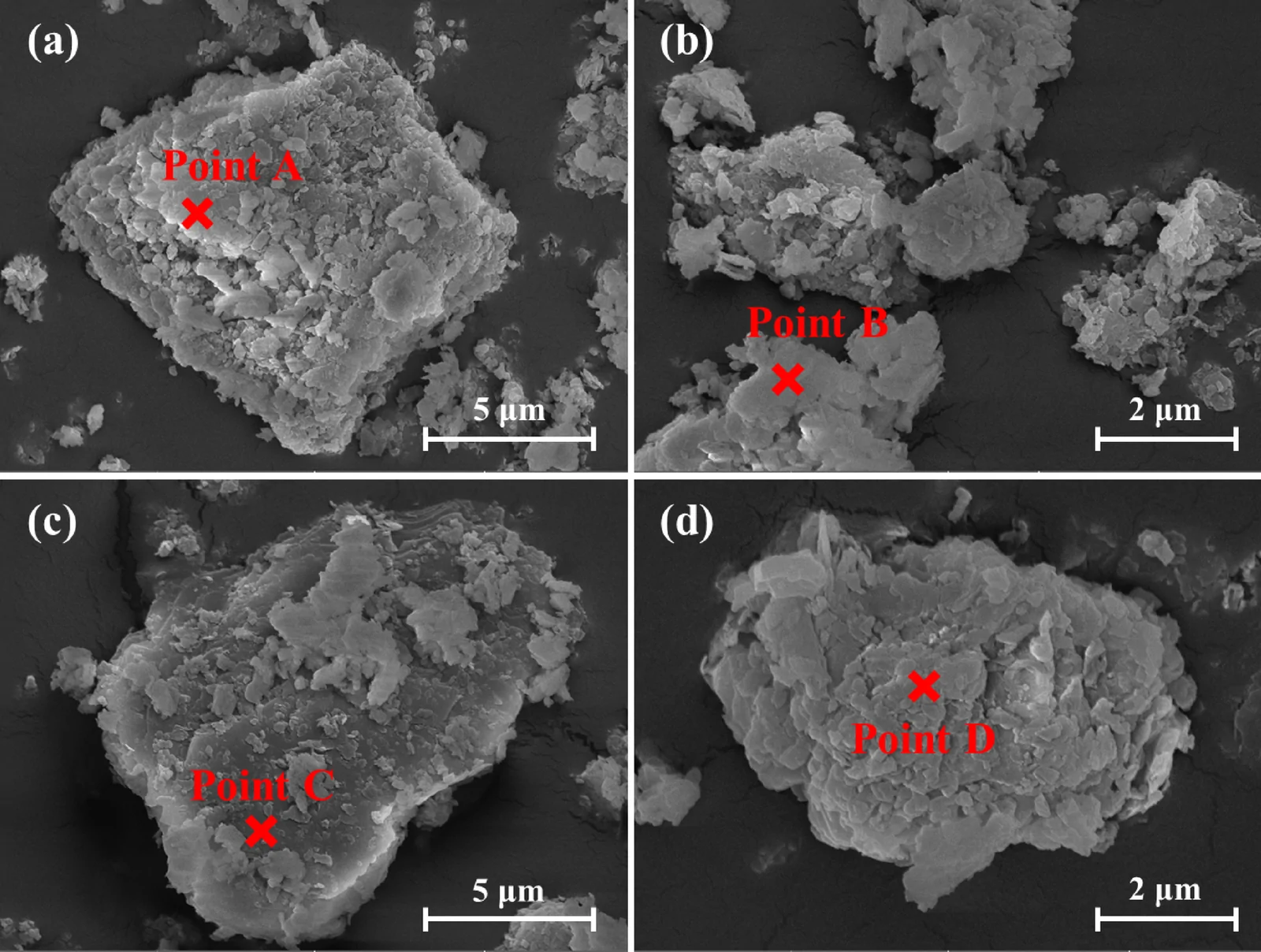
SEM–EDS analysis of (**a**) roasted coal gangue, (**b**) 0.8 mol/L citric acid leaching residue, (**c**) 0.8 mol/L hydrochloric acid leaching residue, and (**d**) 0.032 mol/L hydrochloric acid leaching residue.

The roasted coal gangue and the residues obtained under different leaching conditions were analyzed by SEM–EDS. The morphological images are shown in Fig. 8, and the surface elemental compositions are summarized in Table 3. After roasting pretreatment, the coal gangue still retained the layered structure of kaolinite (Fig. 8a), but its primary mineral phase has been transformed into metakaolinite^29^. It can be seen from Fig. 8 that the leaching residues obtained under different leaching conditions and the roasted coal gangue were similar in their microscopic morphology, all being composed of a layered structure, which indicates that these structures were not significantly disrupted during the acid leaching. However, EDS analysis reveals that under the leaching conditions of 0.8 mol/L citric acid and hydrochloric acid, the Si/Al atomic ratio on the surface of the leaching residues increased to 2.6 and 3.0 (Table 3), respectively. The Si/Al atomic ratio on the surface of the leaching residue obtained using 0.032 mol/L hydrochloric acid was 1.0, consistent with that of roasted coal gangue. Combined with the leaching experimental results, it can be inferred that the leaching of REY by citric acid primarily depends on the disruption of the aluminosilicate structure by H^+^ and the accompanying dissolution of the REY‑bearing phases.

**Table 3:** Elemental composition of the sample determined by SEM–EDS.

Points*	Weight percent (wt%)				Atomic percent (at%)				
	O	Al	Si	Ca	O	Al	Si	Ca	Si/Al
A	39.6	29.0	30.2	1.3	53.2	23.1	23.1	0.7	1.0
B	51.2	13.1	35.7		64.6	9.7	25.6		2.6
C	49.7	12.1	38.1		63.2	9.1	27.6		3.0
D	45.0	27.1	28.0		58.4	20.8	20.7		1.0

* Data from EDS point analyses performed at the locations indicated in Fig. 8.

### Mechanism of REY leaching from roasted coal gangue using citric acid

#### Zeta potential analysis

**Fig. 9:**
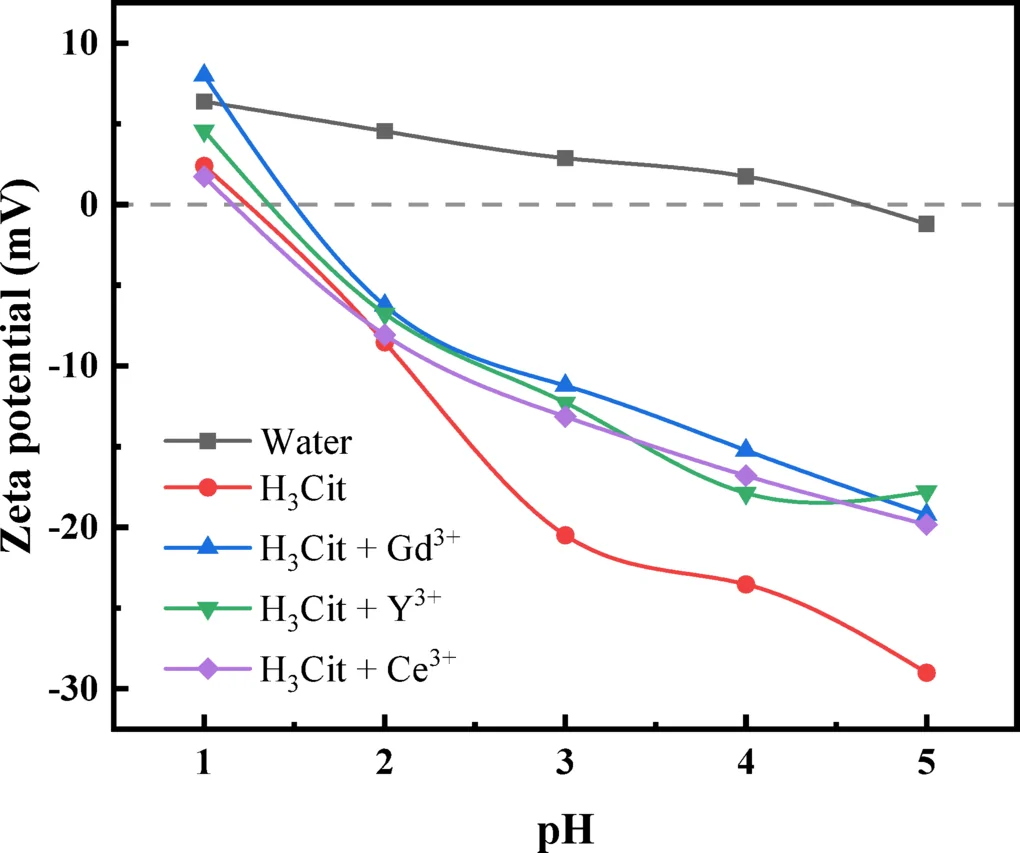
Effect of citric acid and rare earth ions on the zeta potential of roasted coal gangue.

Acid leaching experimental results showed that the leaching behavior of REY differed between citric acid and hydrochloric acid. The adsorption of citric acid on the surface of mineral particles can affect the leaching behavior of REY^33,42^. In this study, electro-kinetic tests were conducted to reveal the impact of citric acid adsorption on the leaching behavior of REY. The results are presented in Fig. 9. Without the addition of citric acid, the zeta potential of the roasted coal gangue decreased as the pH increased, and the point of zero charge was observed at around pH 4.6. In the presence of citric acid, the zeta potential of the roasted coal gangue was lower and decreased rapidly as the pH increased. Meanwhile, the point of zero charge shifted to the left, occurring at approximately pH 1.5. The results indicate that specific adsorption of citric acid occurs on the surface of the roasted coal gangue. Under acidic conditions, citric acid adsorbed on the surface of roasted coal gangue particles exists mainly in its protonated form^32,43^. As the pH increases, the H^+^ concentration decreases, causing citric acid to continuously dissociate and generate more negative charge sites, which significantly enhances the negative charge on the particle surface.

Further, the adsorption behavior of rare earth ions onto the roasted coal gangue surface in the presence of citric acid was examined, as shown in Fig. 9. In the pH range of 1.0–2.0, the presence of Gd^3+^ and Y^3+^ increased the zeta potential of the roasted coal gangue surface, while the presence of Ce^3+^ slightly decreased it. This may be attributed to the high charge density and strong coordination ability of HREY, which render them more readily complexed with citric acid adsorbed onto the surface of the roasted coal gangue^44^. When the pH was higher than 2.0, the zeta potential on the surface of roasted coal gangue was significantly increased in the presence of Ce^3+^, Gd^3+^, and Y^3+^. This phenomenon confirms that specific adsorption of rare earth ions occurs on the surface of roasted coal gangue particles. Therefore, a higher pH environment can promote the complexation reaction between citrate ions and rare earth ions^45,46^. Given that the REY leaching reaction mainly occurs in the pH range of 1.5–2.0 during citric acid leaching, it can be inferred that the adsorption of citric acid on the roasted coal gangue surface has a weak influence on REY leaching. The leached rare earth ions may be retained near the surface of roasted coal gangue by adsorbed citrate ions.

#### Species distribution of REY-Citrate in the leaching solution

Citric acid is one of the stronger acids among low molecular weight organic acids and possesses the chemical characteristics of stepwise dissociation (Eqs. (2–4))^47^. Citric acid aqueous solution has a strong pH buffering capacity. During the leaching reaction with minerals, the pH of the leachate does not change dramatically due to H^+^ consumption and can remain stable at a relatively low initial pH, which is conducive to the leaching of REY. The citric acid leaching process of REY from roasted coal gangue involves many complex reactions, with the main possible reactions shown as Eqs. (5), (6) and (7).

2 $${\mathrm{H}}_{3} {\mathrm{Cit}} \rightleftharpoons {\mathrm{H}}^{ + } + {\mathrm{H}}_{2} {\mathrm{Cit}}^{ - }$$

3 $${\mathrm{H}}_{2} {\mathrm{Cit}}^{ - } \rightleftharpoons {\mathrm{H}}^{ + } + {\mathrm{HCit}}^{2 - }$$

4 $${\mathrm{HCit}}^{2 - } \rightleftharpoons {\mathrm{H}}^{ + } + {\mathrm{Cit}}^{3 - }$$

5 $${\mathrm{Al}}_{2} {\mathrm{O}}_{3} + 6{\mathrm{H}}^{ + } = 2{\mathrm{Al}}^{3 + } + 3{\mathrm{H}}_{2} {\mathrm{O}}$$

6 $${\mathrm{Fe}}_{2} {\mathrm{O}}_{3} + 6{\mathrm{H}}^{ + } = 2{\mathrm{Fe}}^{3 + } + 3{\mathrm{H}}_{2} {\mathrm{O}}$$

7 $${\mathrm{RE}}_{2} {\mathrm{O}}_{3} + 6{\mathrm{H}}^{ + } = 2{\mathrm{RE}}^{3 + } + 3{\mathrm{H}}_{2} {\mathrm{O}}$$

**Fig. 10:**
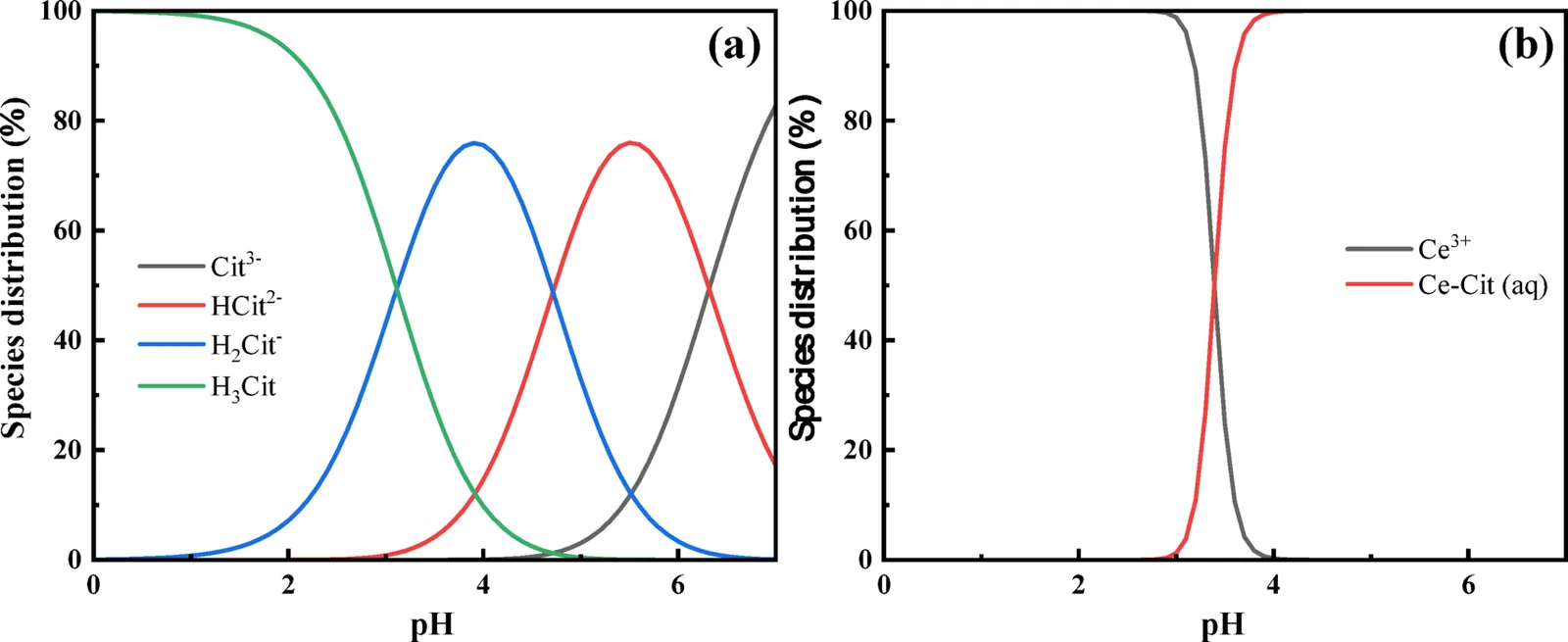
(**a**) Species distribution of citric acid at different pH values (0.8 mol/L, 25 °C). (**b**) Species distribution of Ce in citric acid solution at different pH values (Ce^3+^  = 0.001 mol/L, citric acid = 0.8 mol/L, 25 °C).

In addition, the impact of the complexation ability of citrate ions on REY leaching behavior has been discussed in previous studies^32,33^. Zeta potential analysis indicates that the species of citrate ions in solution have a significant impact on their complexation behavior^32,48^. As the pH of the solution increases, citric acid is sequentially deprotonated, as shown in Fig. 10a. In the optimal citric acid leaching conditions established for this study, the leaching of REY from roasted coal gangue primarily occurs within a pH range of 1.5–2.0. Within this pH range, the citric acid in the solution exists primarily in the form of undissociated H_3_Cit. Figure 10b presents the distribution of the various Ce-containing species in the leachate, as calculated by solution chemical equilibrium. In the citric acid leachate, the REY‑related species are rare earth citrate complexes (Ce-Cit) and Ce^3+^. In the pH range of 1.5–2.0, Ce exists as free Ce^3+^. When the pH exceeds 3, Ce^3+^ rapidly forms complexes with citrate ions. Nevertheless, H_2_Cit^-^ and HCit^2−^ may have weak complexing effects on rare earth ions^48,49^, which is consistent with the results of the zeta potential analysis.

#### Leaching mechanism of REY

**Fig. 11:**
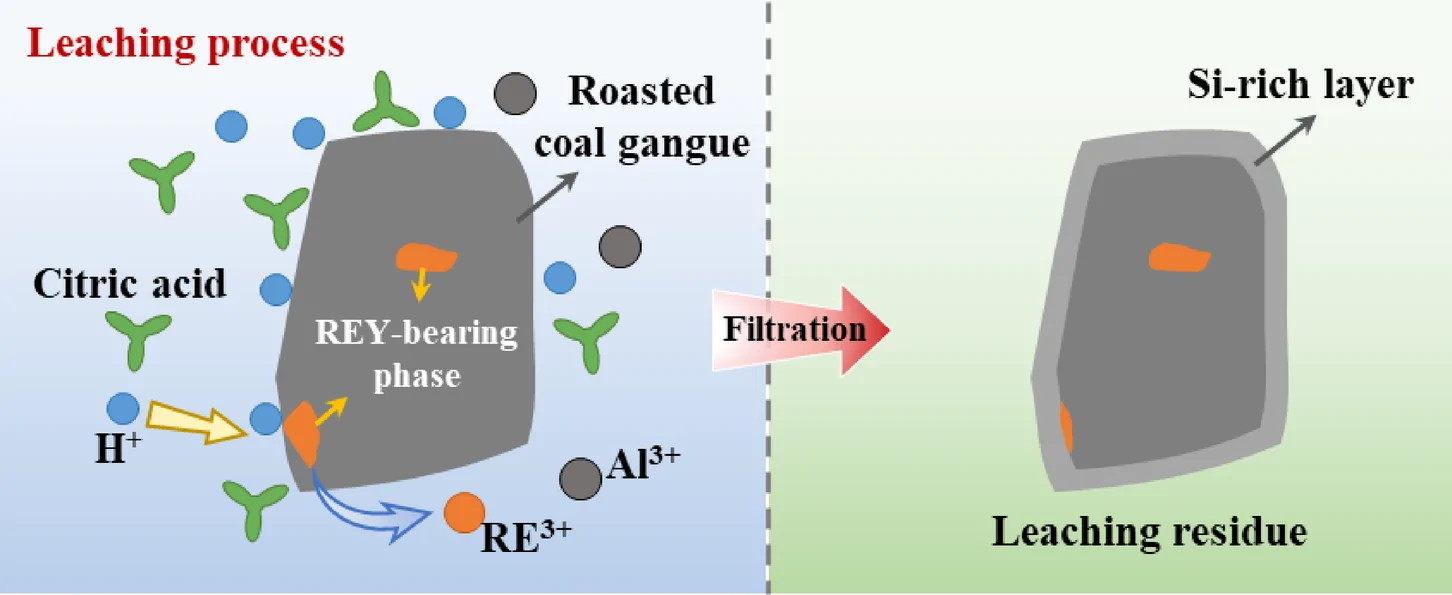
Leaching mechanism of rare earth elements from roasted coal gangue using citric acid.

Based on the above results and our previous studies^17,29^, the leaching mechanism of REY from roasted coal gangue using citric acid is illustrated in Fig. 11. Monazite and bastnaesite are the main carrier minerals of REY in the coal gangue from the Haerwusu mine, and they are embedded within kaolinite^17,40^. After roasting at 600 °C, bastnaesite decomposes into rare earth oxides and rare earth fluorides, while kaolinite transforms into metakaolinite^17^. These mineral phase changes favor the exposure of REY and consequently enhance their leachability. During citric acid leaching, H^+^ is the dominant factor in disrupting the mineral structure and driving REY leaching. The H^+^ released by the dissociation of citric acid preferentially reacts with the Al-O bonds in metakaolinite and the exposed REY on the surface, promoting the migration of Al^3+^ and rare earth ions into the liquid phase. As the leaching reaction progresses, H^+^ continuously diffuses into the interior of the particles, gradually releasing Al and the encapsulated REY, while Si remains on the particle surface, gradually forming a Si‑rich layer. Notably, the leaching reaction mainly occurs in the pH range of 1.5–2.0, where citric acid exists predominantly as H_3_Cit molecules. Zeta potential analysis and solution chemical equilibrium calculations indicate that the complexation of citrate ions with rare earth ions is weak within the pH range of 1.5–2.0. Therefore, the leaching of REY from roasted coal gangue by citric acid is largely governed by the acid dissolution action of H^+^.

### Sustainability of the citric acid leaching process

Coal gangue is the primary solid waste generated during coal mining and processing. Currently, the utilization pathways of coal gangue are largely limited to low-value-added fields such as power generation, construction materials, and ecological backfilling^22^. The recovery of REY from coal gangue holds promise for converting an existing environmental burden into a strategically valuable rare earth resource. Table 4 compares the REY leaching efficiency obtained in this study with those reported in the literature. Although previous studies have reported on the extraction of REY from coal‑based solid wastes using citric acid, most of them have focused on coal fly ash. REY in coal fly ash is encapsulated in the aluminosilicate glass phase^50^, which differs from their occurrence mode in coal gangue. Therefore, the citric acid leaching process for REY recovery from coal gangue still deserves attention. At present, most studies on REY recovery from coal gangue are limited to inorganic acid leaching. Inorganic acids are highly corrosive, easily causing equipment damage and leading to related environmental issues, which is inconsistent with the requirements of sustainable technological development. This study demonstrates that citric acid leaching not only recovers REY from roasted coal gangue but also provides a more environmentally friendly alternative approach compared to conventional inorganic acids.

**Table 4:** Comparison of rare earth elements leaching efficiencies obtained in this study with those reported in the literature.

Sample	Conditions	Leaching efficiency	Reference
Coal ash	Citric acid leaching (5% (w/v), 90 °C, 1 h, 150 mL/g)	About 47% (REY)	Banerjee et al.^34^
Coal fly ash	Multistep NaOH treatment (6 mol/L, 90 °C, 30 min, 10 mL/g) + Citric acid leaching (0.05 mol/L, 75 °C, 2.5 h, 100 mL/g)	55% (REY)	Pan et al.^50^
Coal fly ash	Roasting (800 °C, 2 h, coal fly ash/Na_2_CO_3_ = 1) + Citric acid leaching (0.5 mol/L)	71.5% (REY)	Gao et al.^36^
Coal coarse refuse	Roasting (600 °C, 2 h) + Citric acid leaching (0.05 mol/L, pH = 3, 75 °C, 2 h, 100 mL/g)	About 65% (LREY), about 33% (HREY)	Ji et al.^32^
Coal gangue	Roasting (600 °C, 0.5 h) + HCl leaching (2.0 mol/L, 90 °C, 1 h, 15 mL/g)	78.8% (REY), 82.3% (LREY), 61.3% (HREY)	Chen et al.^17^
Coal gangue	Roasting (600 °C, 2 h) + HCl leaching (0.5 mol/L, 50 °C, 15 min, 50 mL/g)	About 80% (REY)	Wang et al.^27^
Coal gangue	Roasting (600 °C, 2 h) + Citric acid leaching (0.8 mol/L, 90 °C, 150 min, 25 mL/g)	48.6% (REY), 59.1% (LREY), 17.2% (HREY)	This work

From an environmental perspective, the citric acid leaching process offers certain advantages over conventional inorganic acid leaching processes in terms of reducing environmental risks. Citric acid is less corrosive, which helps avoid the hazards associated with splashes and aerosols from strong acids, making the operating environment safer. Moreover, citric acid also possesses excellent biodegradability, which gives its leachate a natural advantage in ecological compatibility during post-treatment, thereby significantly reducing persistent environmental risks. Meanwhile, the citric acid leaching method also has advantages in terms of economic benefits. Under mild conditions at a pH of 1.5–2.0, citric acid leaching can effectively extract REY from roasted coal gangue. This means that leaching equipment can use more conventional materials, reducing investment and maintenance costs. Citric acid in the waste solution can be more easily recovered and regenerated through methods such as precipitation and ion exchange, enabling acid recycling, thereby effectively reducing reagent consumption and waste treatment costs. In summary, the citric acid leaching process exhibits advantages in minimizing hazardous waste generation and reducing secondary pollution risks, which aligns with the green and sustainable development concept of solid waste resource utilization.

## Conclusions

In this work, citric acid leaching experiments were conducted on roasted coal gangue to recover REY. The leaching efficiencies of REY, LREY, and HREY reached 48.6%, 59.1%, and 17.2%, respectively, under conditions of 0.8 mol/L citric acid concentration, a liquid-to-solid ratio of 25, a leaching time of 150 min, and a leaching temperature of 90 °C. Subsequently, the leaching behavior of REY from roasted coal gangue was compared under citric acid and hydrochloric acid conditions. Acid leaching experiments with a fixed concentration of 0.8 mol/L indicated that hydrochloric acid leached REY more rapidly than citric acid. At the same initial pH (approximately 1.5), citric acid showed a higher REY leaching efficiency and the smallest pH change (0.43) before and after leaching. This is mainly because citric acid continuously dissociates to supply H^+^, driving the leaching reaction forward. The citric acid leaching reaction mainly occurs in the pH range of 1.5–2.0. Meanwhile, the Al leaching efficiency was 51.0% under 0.8 mol/L citric acid conditions. SEM–EDS analysis shows that the Si/Al atomic ratios of the residues from 0.8 mol/L citric acid and hydrochloric acid leaching were 2.6 and 3.0, much higher than the atomic ratio of 1.0 for the roasted coal gangue. Zeta potential analysis reveals that citric acid can adsorb onto the surface of roasted coal gangue, thereby influencing the leaching of REY. Solution chemical equilibrium calculations indicate that the complexation of citrate ions with rare earth ions is weak during the leaching stage. These findings suggest that the leaching of REY from roasted coal gangue by citric acid is mainly attributed to the acid dissolution effect of H^+^. This study can provide guidance for optimizing the REY recovery processes from coal gangue under mild acid conditions.

## Data Availability

The data will be made available from the corresponding author upon reasonable request.
